# PReS-FINAL-2348: Identification of autoantibodies against inner ear antigens in a cohort of children with idiopathic sensorineural hearing loss

**DOI:** 10.1186/1546-0096-11-S2-P338

**Published:** 2013-12-05

**Authors:** G Vannucci, E Berti, G Simonini, C Lunardi, C Bason, T Giani, I Pagnini, D Moretti, E Marrani, R Cimaz

**Affiliations:** 1 Rheumatology Unit, Anna Meyer Children Hospital, Florence, Italy; 2 Department of Clinical and Experimental Medicine, G.B. Rossi Hospital and University of Verona,, Verona, Italy

## Introduction

Immune-mediated pathogenesis has been suggested for idiopathic sensorineural hearing loss (iSNHL). Recent studies have investigated the relationship between iSNHL and autoantibodies against inner ear antigens, but specific tests are not available.

## Objectives

To assess the positivity of autoantibodies against inner ear in a series of pediatric patients with idiopathic sensorineural hearing loss.

## Methods

We conducted a prospective, non-interventional observational study in a series of pediatric patients affected with iSNHL. Autoantibodies against inner ear (anti-Cogan peptide, anti-connexin 26, anti-DEP1/CD148 and anti-reovirus), previously described in the serum of patients with Cogan's syndrome, were detected in our populations. The characteristics of children who resulted positive were also evaluated to verify if clinical data, disease progression and response to treatment could confirm an immune-mediated pathogenesis.

## Results

Eleven patients were enrolled and 9 of them resulted positive for the inner ear antibodies detected. Non-organ specific autoantibodies were present in 5 children out of 9. An immune-mediated condition was diagnosed in 2 cases and minor immune manifestations were found in other 2 patients. In 5 cases hearing loss remained stable with no therapy, otherwise 4 children developed hearing loss progression. Two subjects were treated with steroids and methotrexate achieving hearing improvement. Another subject started methotrexate treatment showing hearing loss stabilization.

## Conclusion

Most of clinical characteristics and comorbidities described, added to immunologic tests results and to treatment efficacy, suggest an immune-mediated pathogenesis of hearing loss. Inner ear autoantibodies are not able to identify children affected with autoimmune sensorineural hearing loss but, integrated with clinical data, they can represent a diagnostic aid for the physician. Large prospective studies are needed to investigate usefulness, diagnostic and prognostic role of these autoantibodies.

## Disclosure of interest

None declared.

